# Supplementary material for: Multi-Trait Genomic Prediction of Yield-Related Traits in US Soft Wheat under Variable Water Regimes
Source: Genes (Basel). 2020 Oct 28;11(11):1270. doi: 10.3390/genes11111270 (PMC7716228; doi:10.3390/genes11111270)
Supplement: Supplementary file 1 [file genes-11-01270-s001.pdf]

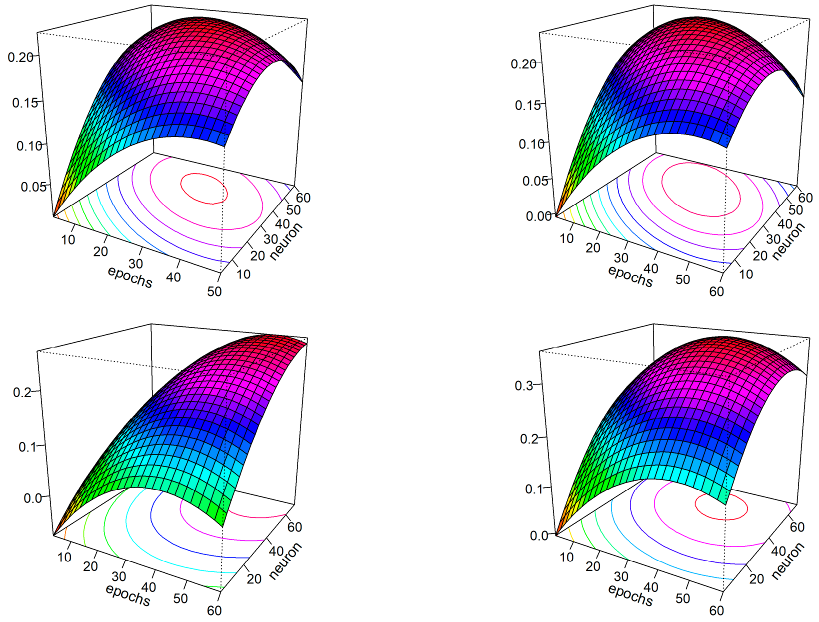

(**a**) MMDL not stratified

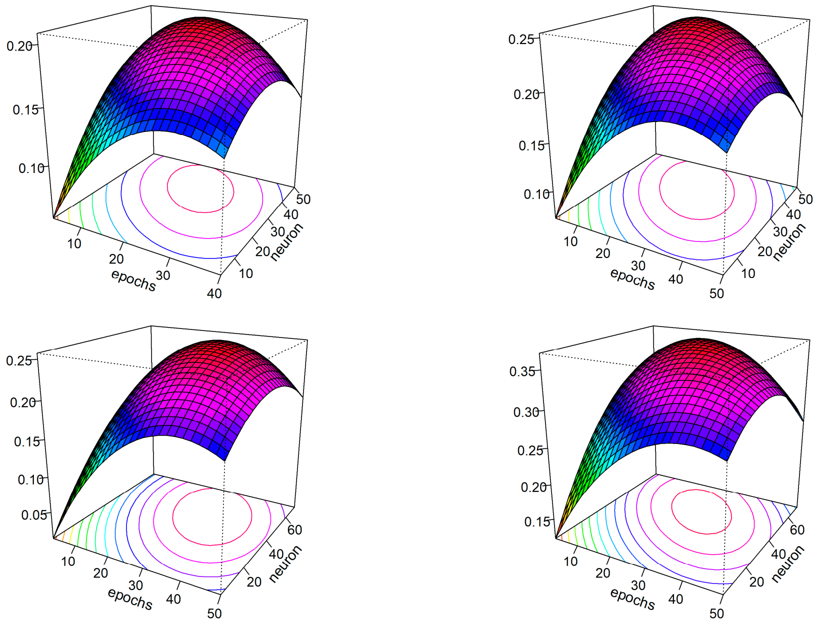

(**b**) SMDL not stratified

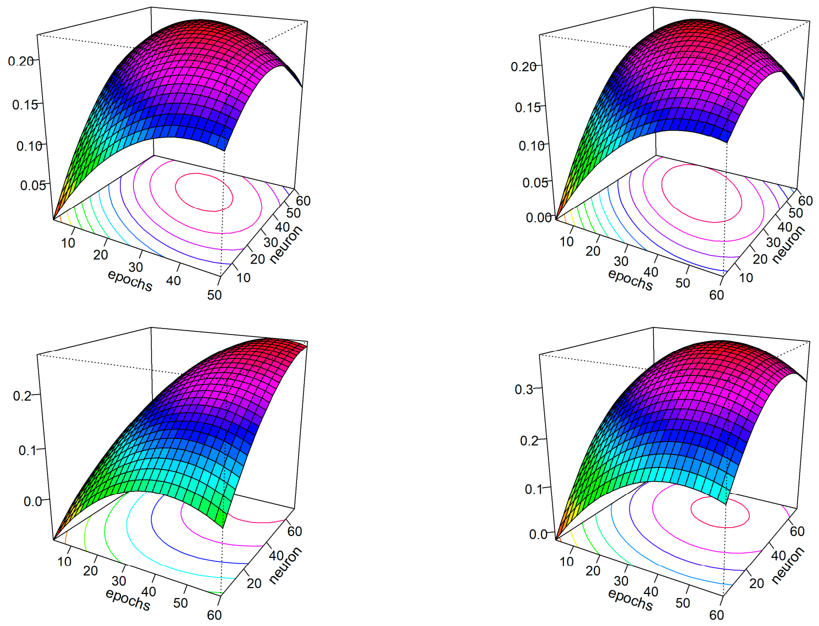

(**c**) MMDL stratified

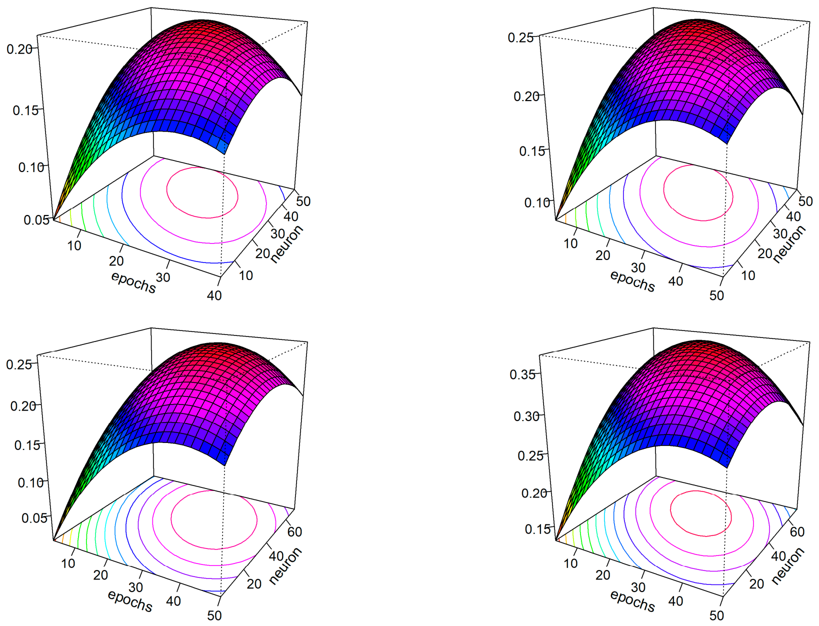

(**d**) SMDL stratified

Figure S1. Fitted second-order response surface plots near the stationary point for optimal epoch and neuron combinations for prediction accuracy of grain yield, harvest index, spike fertility, and thousand grain weight averaged over four environments. The analysis was performed for each model including (a) un-stratified Multi-trait Multi-environment Deep Learning, (b) un-stratified Single-trait Multi-environment Deep Learning, (c) stratified Multi-trait Multi-environment Deep Learning, and (d) stratified Single-trait Multi-environment Deep Learning model. The *y*-axis value represents the averaged Pearson's correlation between observed and predicted phenotypic value. The two *x*-axis values represent the number of epochs and neurons, respectively. The optimal point was located by investigating the stationary point for each response surface.
